# Experimental and Machine Learning Modelling of Ni(II) Ion Adsorption onto Guar Gum: Artificial Neural Network (ANN) and K-Nearest Neighbor (KNN) Comparative Study

**DOI:** 10.3390/polym17202791

**Published:** 2025-10-18

**Authors:** Ismat H. Ali, Malak F. Alqahtani, Nasma D. Eljack, Sawsan B. Eltahir, Makka Hashim Ahmed, Abubakr Elkhaleefa

**Affiliations:** 1 Chemistry Department, College of Science, King Khalid University, Abha 61314, Saudi Arabia; angel.ihali@kku.edu.sa; 2 Department of Chemistry, Turaba University College, Taif University, Taif 21944, Saudi Arabia; ndmansouri@tu.edu.sa; 3 Department of Chemistry, Faculty of Science, University of Khartoum, Khartoum 11115, Sudan; 4 Department of Chemistry, College of Science, University of Hafr Al Batin, Hafr Al Batin 39524, Saudi Arabia; sawsanbt@uhb.edu.sa (S.B.E.); mhnasr@uhb.edu.sa (M.H.A.); 5 Department of Chemistry, Faculty of Science, Sudan University for Science and Technology, Khartoum 11116, Sudan; 6 Department of Chemical Engineering, College of Engineering, King Khalid University, Abha 61411, Saudi Arabia; amelkhalee@kku.edu.sa

**Keywords:** adsorption, guar gum, Ni(II) ions, artificial neural network, kinetics

## Abstract

In this study, a guar gum-based adsorbent was developed and evaluated for the removal of Ni(II) ions from aqueous solutions through a combined experimental and machine learning (ML) approach. The adsorbent was characterized using FTIR, SEM, XRD, TGA, and BET analyses to confirm surface functionality and porous morphology suitable for metal binding. Batch adsorption experiments were conducted to optimize the effects of pH, adsorbent dosage, contact time, temperature, and initial metal concentration. The adsorption efficiency increased with higher pH and adsorbent dosage, achieving a maximum Ni(II) removal of 97% (qₘ = 86.0 mg g^−1^) under optimal conditions (pH 6.0, dosage 1.0 g L^−1^, contact time 60 min, and initial concentration 50 mg L^−1^). The process followed the pseudo-second-order kinetic and Langmuir isotherm models. Thermodynamic results revealed the spontaneous, endothermic, and physical nature of the adsorption process. To complement the experimental findings, artificial neural network (ANN) and k-nearest neighbor (KNN) models were developed to predict Ni(II) removal efficiency based on process parameters. The ANN model yielded a higher prediction accuracy (R^2^ = 0.97) compared to KNN (R^2^ = 0.95), validating the strong correlation between experimental and predicted outcomes. The convergence of experimental optimization and ML prediction demonstrates a robust framework for designing eco-friendly, biopolymer-based adsorbents for heavy metal remediation.

## 1. Introduction

Heavy metals in wastewater and/or drinking water are among the riskiest environmental problems. This problem is likely due to the dumping of crude industrial wastes [1,2,3,4]. Various industries involve a final treatment process consisting of metal compounds that can cause contamination in the discharged water [2,5,6]. Most of these heavy metals are non-biodegradable, having a long biological half-life, leading to potential accumulation and human exposure through food or water [1]. Nickel ions are commonly found in water in the form of oxides, nitrates, and sulfides. However, when their concentration exceeds safe limits, they can lead to serious health problems, e.g., skin dermatitis, pulmonary fibrosis, nausea, vomiting, and even neurological disorders in children [3,6,7,8,9]. Traditional methods for removing nickel from wastewater, such as coagulation, ion exchange and chemical precipitation, are often costly and generate large amounts of toxic sludge [10]. In response, researchers have recently turned to low-cost, renewable, and eco-friendly agricultural and natural materials as promising alternatives for nickel removal through biosorption [11,12,13,14].

Guar gum is composed of polysaccharides. Guar gum is made up of a backbone of mannose sugars linked in a straight chain, with galactose sugars branching off at every other mannose unit, forming short side chains. Guar gum is also known for its ability to withstand temperatures of up to 80 °C for several minutes without losing its stability. This composition helps Guar gum be an effective adsorbent for removing organic and inorganic pollutants from wastewater [4,10]. The effectiveness of this low-cost adsorbent is fundamentally due to the functional groups present in the polysaccharide structure [10].

Nickel(II) ions present in wastewater can be effectively removed using a variety of adsorbents [11,12,13,14]. Studies have shown that the efficiency of this process depends greatly on the operating conditions, particularly the initial concentration of metal ions, the dosage of the adsorbent, and the pH of the solution. Akram et al. investigated Ni(II) removal using a magnesium–Punica granatum Linn-based adsorbent, achieving a maximum capacity of 45 mg·g^−1^ and 97% efficiency. The study, however, was limited to a single temperature (318 K) and lacked AI-based modeling, reducing its comparative depth and analytical scope [12]. Nanobentonite has been reported to effectively remove Ni(II) and Cu(II) ions from aqueous solutions within a concentration range of 50–300 mg·L^−1^, following the Langmuir isotherm and pseudo-second-order kinetics; however, the study lacked any artificial intelligence (AI)-based modeling, which could have enhanced prediction accuracy and process optimization [15].

An artificial neural network (ANN) is a learning tool that mimics the human mind, which is made up of an input layer, one or more hidden layers, and an output layer. Each of these layers contains many nodes, often called neurons, that are connected to one another and work together to process and transmit information through the network. ANN is used to predict adsorption behavior based on experimental data. Each layer uses the preceding layer as its input to form an interconnected neural network. The neurons number in the input layer depends on the parameters used in the study [16]. ANN has been used in many applications to predict adsorption behavior and verify its efficiency successfully, such as using experimental data for the decontamination of Cr(VI) by ferrochrome slag/polyaniline adsorbent, where the model used gave the maximum correlation coefficient (R = 0.991) and lowest mean square error (MSE = 9.801) [17]. ANN was used to predict the adsorption efficiency of Ni(II) with perlite in aqueous solutions using 140 experimental data sets. The model consists of three neurons in the input layer, viz. contact time, total concentration, and adsorbent mass. The results exhibited that the ANN prediction data is in good agreement with the experimental data [18].

This study aims to characterize, use, evaluate, and compare the adsorption of Ni(II) ions by Guar Gum (GG) without any thermal or/and chemical treatment. The parameters affecting adsorption efficiencies, such as pH, GG mass, contact time, initial Ni(II) ion concentration, and temperature, were studied. To our knowledge, GG has not previously been reported for removing Ni(II) ions from synthetic wastewater. Finally, a model containing a training algorithm to predict removal efficiency using ANN is created and verified by calculating Mean Square Error (MSE) and Correlation Coefficient (R^2^), where the results of the study indicate how closely the experimental data match the predicted values.

## 2. Materials and Methods

### 2.1. Chemicals and Instruments

All chemicals used in this study were obtained from BDH (Bradford, UK) and Merck (Darmstadt, Germany) and were used without further purifications. In deionized water, Ni(II) ion stock solutions were prepared from nickel nitrate. Several Ni(II) ion concentrations were prepared by diluting the stock solutions. HCl and NaOH solutions were employed to adjust the pH value. The pH tests were performed using a pH meter Hanna 211 (Woonsocket, RI, USA). An atomic absorption spectrometer determined the equilibrium Ni(II) ion concentrations in the treated solutions Spectra AA 20 (Varian Inc., Palo Alto, CA, USA).

### 2.2. Sample Characterization

The composition of GG was analyzed using an ATR spectrometer (Perkin Elmer, Springfield, IL, USA). The crystallinity of the guar gum (GG) samples was determined with an X-ray powder diffractometer (Japanese Dmax-rA, CuKα radiation, λ = 1.54 Å), scanned over the 2θ range of 80–5° with a step size of 2%. The surface morphology of GG was examined using Scanning Electron Microscopy (SEM) (FEI Company, Eindhoven, The Netherlands) operated at 20 kV accelerating voltage. Furthermore, the surface area of GG was measured with a Micromeritics ASAP 2020 Surface Area and Porosity Analyzer (Atlanta, GA, USA) following the Brunauer–Emmett–Teller (BET) method. Thermogravimetric analysis (TGA) was carried out using a TA Instruments Q50 analyzer (New Castle, DE, USA) under N_2_ atmosphere (50 mL·min^−1^) with a heating rate of 10 °C·min^−1^ from 25 °C to 800 °C under a continuous nitrogen atmosphere.

### 2.3. Adsorption Experiments

A stock solution of Ni(II) ions (1000 ppm) was first prepared, and the chosen concentrations were prepared by diluting it with deionized water. For each batch experiment, a measured amount of guar gum (GG) was mixed with 50 mL of Ni(II) solution (adjusted to the required pH) in plastic bottles. The mixtures were then placed on a shaker and allowed to equilibrate at a controlled temperature for predetermined time intervals. Each experiment was carried out in duplicate; the plotted data represent mean values of the two measurements. The oscillation rate of the shaker was 150 rpm. The quantity of Ni(II) ions adsorbed by GG was determined using Equation (1).

(1) $$q_{e}=\frac{{(C}_{i}-C_{e})V}{m}\;\;\;$$

Here, *qe* represents the adsorption capacity (mg g^−1^); *C*_*i*_ and *C*_*e*_ are the initial and equilibrium concentrations of Ni(II) (mg L^−1^); V is the volume of the solution (L); and m is the GG mass (g).

To explore the effect of the initial pH value, the required mass of GG was mixed with 50 mL of known concentration of Ni(II) solution in batch experiments with the pH values kept between 2 to 11 using HCl and/or NaOH. The experiments were performed at 25 °C. Upon equilibration, the residual Ni(II) concentration was assessed using an atomic absorption spectrometer. The removal percent (R%) of Ni(II) ions from aqueous solutions was determined by Equation (2).

(2) $$R\%=\frac{\left(C_{i}-C_{e}\right)}{C_{i}}\times 100\;\;\;\;$$

The thermodynamic parameters were calculated using several experiments conducted at a 20–40 °C temperature range. Kinetic experiments were performed by studying Ni(II) adsorbed amounts as a function of contact time (10–60 min). Adsorption isotherms were obtained following the same batch procedure, with initial Ni(II) concentrations varied between 50 and 400 ppm. After equilibration, the mixtures were filtered, and the residual Ni(II) concentrations were subsequently determined.

The selectivity of GG adsorbent was evaluated using aqueous solutions containing a mixture of Cr(VI), Cu(II), Pb(II), and Ni(II) ions, each at an initial concentration of 50 mg L^−1^. A mass of 0.80 g of GG was added to 50 mL of the mixed-ion solution, adjusted to pH 6.0, and the suspensions were equilibrated on a shaker for 40 min at 25 °C. The residual concentration of Ni(II) in the aqueous phase was measured using atomic absorption spectrophotometry (AAS). It should be noted that AAS detects only the elemental nickel ions (Ni(II) present in solution, not the total salt concentration. The distribution coefficients (D, L g^−1^) and the selectivity coefficients (βNi^2+^/Mn^2+^) were subsequently calculated according to the following equations.

(3) $$D=\frac{\left(C_{i}-C_{f}\right)}{C_{f}}\times \frac{V}{M}\;\;\;\;\;\;$$

(4) $$\beta_{{Ni}^{2+}/M^{n+}}=\frac{D_{{Ni}^{2+}}}{D_{M^{n+}}}\;\;\;$$

For reactivation, the Ni(II)-loaded GG sample was immersed in 100 mL of 0.5 M acidified thiourea solution and stirred for 2 h. The sample was then thoroughly rinsed with deionized water until free from residual acid. The reactivation efficiency was subsequently evaluated using Equation (5).

(5) $$Regeneration\;efficiency\;\%=\frac{amount\;of\;Ni\left(II\right)ions\;adsorbed\;during\;the\;second\;run}{amount\;of\;Ni\left(II\right)ions\;adsorbed\;during\;the\;first\;run}\times 100$$

### 2.4. Reliability of Results

A calibration curve was established for Ni(II) ions within the concentration range of 0.5–5.0 mg·L^−1^. The linearity of the calibration plot was examined to evaluate the reliability and analytical performance of the method. The limits of detection (LOD), quantification (LOQ), and method precision were determined according to the procedure described previously [19]. The analytical accuracy was confirmed through recovery studies.

### 2.5. Artificial Neural Networks (ANNs) for Ni(II) Ion Adsorption

A neural network model was developed in Maple 6 software to predict the removal efficiency of Ni(II) ions from aqueous solutions. The network comprised three layers: an input layer with five parameters (initial Ni(II) concentration, guar gum (GG) mass, pH, contact time, and temperature), a hidden layer with a variable number of neurons, and an output layer with a single neuron representing Ni(II) removal efficiency. Each neuron carried an adjustable weight, and the layers were interconnected such that outputs from one layer served as inputs to the next.

The dataset (42 points) was divided into 70% for training and 30% for testing. A custom learning algorithm was used. The optimal architecture comprised one hidden layer with nine neurons, selected after testing neuron counts from 5–12 using cross-validation to minimize RMSE. The tansig transfer function was applied to the hidden layer and purelin to the output layer. Training used the Levenberg–Marquardt algorithm with early stopping after six epochs without validation improvement. Its predictive accuracy was evaluated by comparing outputs with experimental values, with performance verified through mean square error (MSE) and the correlation coefficient (R^2^), as shown in Equations (6) and (7).

(6) $$MSE=\frac{1}{n_{T}}\sum_{i=1}^{n_{T}}{\left(p_{i}-a_{i}\right)}^{2}$$

(7) $$R^{2}=1-\sum_{i=1}^{n_{T}}\frac{{\left(a_{i}-P_{i}\right)}^{2}}{{\left(a_{i}-{\underline{a}}_{i}\right)}^{2}}$$

In these equations, *a_i_* denotes the experimental value, ${\underline{a}}_{i}$ represents the average of the experimental data, $p_{i}$ is the predicted value, and *nT* refers to the total number of training samples.

### 2.6. K-Nearest Neighbors (KNN) for Ni(II) Ions Adsorption

The K-nearest neighbor (KNN) algorithm was applied to validate the ANN predictions and provide a comparative non-parametric modeling approach. KNN was implemented in MATLAB R2023b using the same dataset and input parameters as the ANN model. Data were normalized between 0 and 1 prior to model training. The optimal value of *k* = 3 was determined using five-fold cross-validation, which minimized prediction error across multiple k-values (1–10). The Euclidean distance metric was used to measure similarity between data points, and the output (Ni(II) removal efficiency) was estimated as the mean of the nearest neighbors.

Model accuracy and generalization were evaluated using R^2^, RMSE, and MAE, following the same evaluation metrics as the ANN model. Comparative analysis demonstrated that both models performed well; however, the ANN model exhibited slightly higher accuracy and lower prediction error, indicating its superior ability to capture nonlinear relationships among adsorption variables. The KNN figure was generated by ChatGPT5.

### 2.7. Model Comparison and Validation

The predictive performance of both models was compared under identical conditions. The ANN model yielded slightly higher R^2^ and lower RMSE and MAE than KNN, confirming its better capability in capturing the nonlinear adsorption behavior. The close agreement between experimental and predicted results demonstrated good model generalization and reliability.

## 3. Results and Discussion

### 3.1. Characterization of GG Adsorbent

#### 3.1.1. Surface Area of GG

Results showed that the average pore diameter is 97 A°, confirming that GG has a mesoporous structure. Moreover, results also indicate that GG has a medium surface area (24.0 m^2^/g) compared to other adsorbents used to adsorb Ni(II) ions [14]. The total pore volume was determined as 23.3 × 10^−2^ cm^3^/g. The BET surface characteristics were measured using dry powdered guar gum, as the material dissolves in water; hence, the reported values represent its dry-state porosity and surface structure.

#### 3.1.2. FTIR Analysis of GG

**Figure 1:**
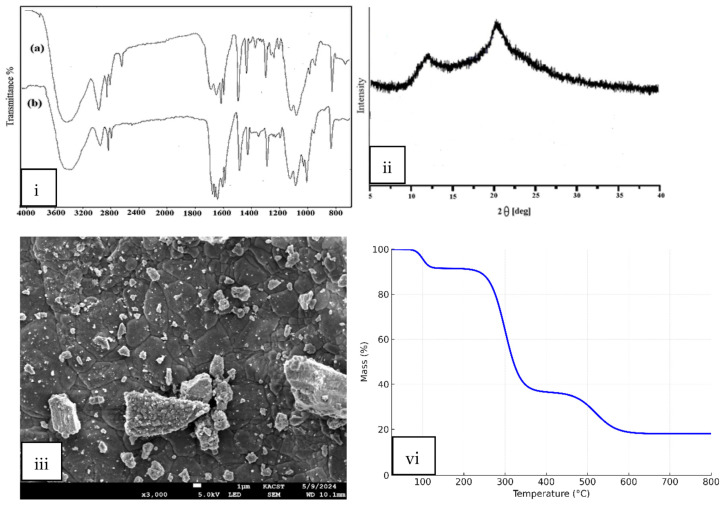
Characterization of guar gum adsorbent: (**i**) FTIR spectra (a) before and (b) after Ni(II) adsorption, (**ii**) XRD patterns of GG, (**iii**) SEM image of GG, and (**vi**) TGA curve of GG.

The FTIR spectra of the adsorbent before and after Ni(II) loading are shown in Figure 1i. The spectrum of the pristine material (spectrum a) displays the characteristic absorption bands of GG, thereby confirming the identity of the adsorbent. A broad band at 3400–3450 cm^−1^ corresponds to O–H stretching vibrations of hydroxyl groups, which are abundant in the galactomannan backbone of GG. The peaks in the region 2920–2850 cm^−1^ are assigned to C–H stretching of aliphatic groups. Absorption at ~1650 cm^−1^ can be attributed to adsorbed water bending vibrations and possible C=O stretching of residual acetyl groups, while the bands in the region 1150–1020 cm^−1^ are connected with C–O–C and C–O stretching vibrations in the polysaccharide structure. The presence of these functional groups (–OH, –C–H, and –C–O–C) is consistent with the chemical composition of Guar Gum, confirming the polymeric adsorbent nature [20].

After Ni(II) adsorption (spectrum b), notable changes are observed, confirming the successful binding of metal ions. The broad –OH stretching band around 3400 cm^−1^ shows a clear reduction in intensity and a slight shift, indicating involvement of hydroxyl groups in metal complexation through hydrogen bonding or direct coordination. The band near 2920 cm^−1^ (C–H stretching) is slightly perturbed, reflecting structural adjustment in the GG matrix upon Ni(II) loading. The absorption band observed at 1630–1650 cm^−1^ in the FTIR spectrum of guar gum is attributed to the C=O stretching vibration of residual carbonyl or carboxyl groups. Although guar gum is primarily a galactomannan polysaccharide composed of mannose and galactose units, minor quantities of uronic acid residues and other oxidized carbohydrate groups are naturally present, which account for the occurrence of this carbonyl band. The presence of this peak, therefore, reflects the inherent chemical composition of guar gum rather than external impurities. After Ni(II) adsorption, the shift and reduction in intensity of this band confirm the participation of these oxygen-containing functional groups in metal ion coordination. Furthermore, in the fingerprint region (1200–1000 cm^−1^), noticeable variations in intensity and shape are observed, confirming that the C–O and C–O–C bonds participate in coordination with Ni(II) ions.

Taken together, the spectral changes provide strong evidence that GG was successfully used as the adsorbent and that its hydroxyl and ether functional groups play a dominant role in Ni(II) uptake. The comparative FTIR spectra thus validate both the identity of GG and the efficient adsorption of Ni(II) ions onto its surface.

#### 3.1.3. X-Ray Diffraction of GG

The XRD pattern of Guar Gum (GG) (Figure 1ii) displays a broad halo with a main peak around 2θ ≈ 20°, which can be indexed to the (110) reflection of polysaccharide-based biopolymers. The absence of sharp and well-defined peaks such as (001) or (100) confirms the predominantly amorphous structure of GG. This amorphous nature, with only weak ordering of molecular chains, is typical of natural polysaccharides and provides numerous disordered sites and functional groups that can participate in adsorption. Such structural features are advantageous, as they facilitate the binding of Ni(II) ions by increasing the availability of active sites [21].

#### 3.1.4. SEM Analysis of GG

The SEM micrograph of Guar Gum (GG) (Figure 1iii) reveals an irregular and heterogeneous surface morphology. The material shows a rough texture with aggregated particles of varying sizes distributed across the surface. The presence of cracks and pores indicates a non-uniform and porous surface, which increases the available surface area for adsorption. Larger agglomerates appear embedded in a continuous matrix, while numerous fine particles are scattered on the surface. Such morphological features are typical of natural polysaccharides and are advantageous for adsorption, as they provide multiple active sites for interaction with Ni(II) ions.

#### 3.1.5. TGA Analysis of GG

The thermogravimetric curve of Guar Gum (GG) (Figure 1vi) shows a three-step degradation profile typical of polysaccharide-based biopolymers. The initial weight loss below 150 °C corresponds to the evaporation of physically adsorbed moisture, accounting for about 8–10% of the total mass. The second major stage, occurring between 220–360 °C, represents the thermal decomposition of the polymer backbone, primarily due to cleavage of glycosidic linkages and breakdown of the galactomannan structure, leading to a mass loss of ~55%. A slower degradation step is observed in the range of 400–700 °C, which is attributed to the carbonization and degradation of more stable residues, leaving about 15–20% char at 800 °C. The FTIR spectra and physical observation confirmed that guar gum retained its structural integrity after repeated adsorption–desorption cycles, indicating minimal degradation during the experimental period.

### 3.2. Adsorption Optimization

#### 3.2.1. pH Effect on Adsorption Efficiency

**Figure 2:**
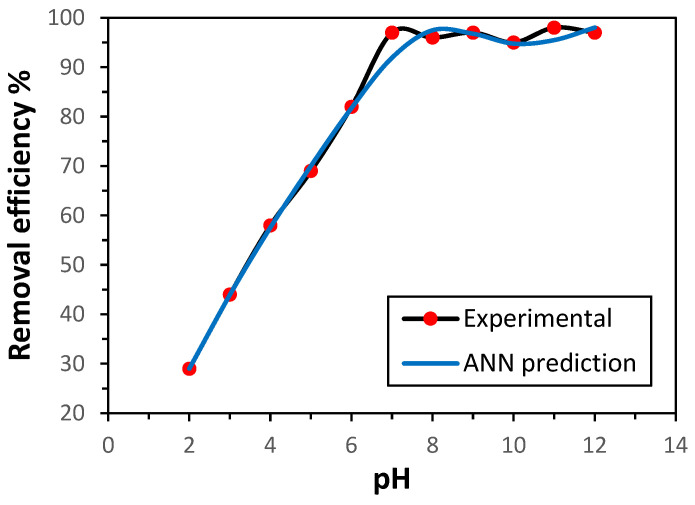
Influence of pH on the adsorption of Ni(II) by GG under fixed conditions (initial concentration 50 mg L^−1^, 0.5 g adsorbent, 30 min contact time, 150 rpm, 25 °C).

The efficiency of adsorption is strongly influenced by the pH value of the medium, as it governs the binding interface between metal ions and the adsorbent surface. In this study, the performance of GG in removing Ni(II) ions was investigated across the pH range of 2–12, using both experimental data and ANN predictions. The ANN-predicted values exhibited good agreement with the experimental results, as illustrated in Figure 2. As anticipated, the adsorption effectiveness was significantly influenced by the solution pH. The removal efficiency increased with rising pH up to 7, beyond which it remained nearly constant. This behavior can be attributed to the competition between Ni^2+^ and H^+^ ions for active adsorption sites on the GG surface under acidic conditions [14]. At pH values above 7, fewer H^+^ ions exist, and therefore more adsorption positions are accessible to Ni(II) ions. The optimal pH value was 7, which was employed throughout this work.

#### 3.2.2. Influence of GG Mass on Adsorption Efficiency

**Figure 3:**
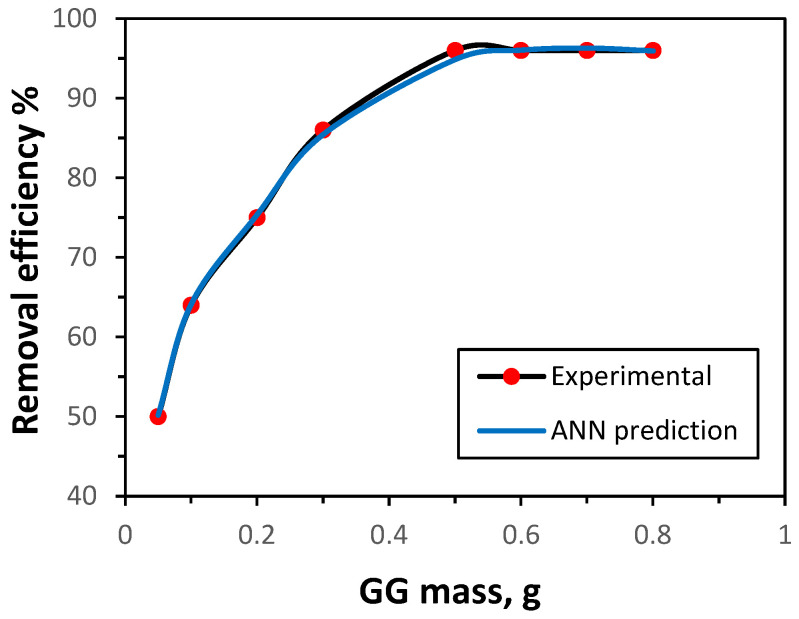
Effect of GG dosage on the uptake of Ni(II) ions (initial concentration 50 mg/L, time 30 min, pH 7.0, shaking rate 150 rpm, 25 °C).

The influence of GG mass on adsorption efficiency was examined to identify the optimum mass. As shown in Figure 3, the removal of Ni(II) ions, based on both experimental data and ANN predictions, increased steadily with GG mass from 0.05 g to 0.50 g. Further increases beyond 0.50 g produced no significant improvement in adsorption efficiency. This trend is consistent with the expectation that a larger adsorbent mass provides a greater surface area and number of active sites, thereby enhancing removal up to a saturation point. The ANN model successfully reproduced this behavior, accurately predicting the adsorption performance. Accordingly, an optimal GG mass of 0.50 g was employed in subsequent experiments.

#### 3.2.3. Influence of Temperature on Adsorption Efficiency

**Figure 4:**
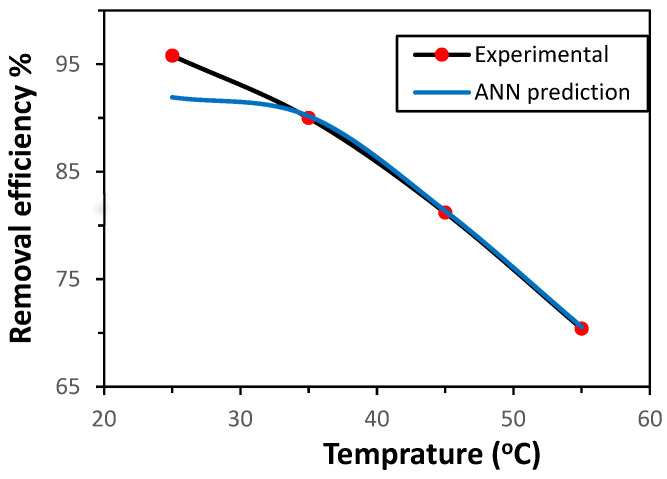
Effect of temperature on the uptake of Ni(II) ions (initial concentration 50 mg/L, adsorbent mass 0.5 g, time 30 min, pH 7.0, shaking rate 150 rpm.

The influence of temperature on Ni(II) adsorption by the GG adsorbent was investigated within the range of 25–55 °C, under the previously established optimal conditions. The removal efficiency was also predicted using the ANN model. As illustrated in Figure 4, the adsorption effectiveness decreased with increasing temperature, indicating that the adsorption process is exothermic. This performance can be ascribed to the weak interactions between Ni(II) ions and the active sites of GG, as well as between adjacent molecules in the adsorbed phase [22]. In addition, the possible deactivation or damage of adsorption sites at elevated temperatures may also contribute to the decline in efficiency [14]. The ANN model demonstrated strong predictive capability, closely matching the experimental results for Ni(II) removal from aqueous solution.

The adsorption kinetics of Ni(II) ions were examined by applying both the pseudo-first-order and pseudo-second-order models to the experimental data. The pseudo-first-order kinetic equation is presented in Equation (8) [23], while the pseudo-second-order kinetic model is expressed in Equation (9) [24].

(8) $$ln\left(q_{e}-q_{t}\right)=lnq_{e}-k_{1}t\;\;\;$$

(9) $$\frac{t}{q_{t}}=\frac{1}{k_{2}q_{e}^{2}}+\frac{t}{q_{e}}\;$$

In these equations, *q*_*e*_ and *q*_*t*_ (mg g^−1^) denote the amounts of Ni(II) ions adsorbed per unit mass of adsorbent at equilibrium and at time *t*, respectively. The parameter *k*_1_ (min^−1^) represents the pseudo-first-order rate constant, whereas *k*_2_ (g mg^−1^ min^−1^) refers to the pseudo-second-order rate constant of the adsorption process.

Linearized plots were used to assess the suitability of the kinetic models, and the calculated parameters are summarized in Table 1. Based on the correlation coefficient (R^2^) values, the adsorption data showed a stronger agreement with the pseudo-second-order model.

#### 3.2.4. Influence of Contact Time on Adsorption Efficiency

**Figure 5:**
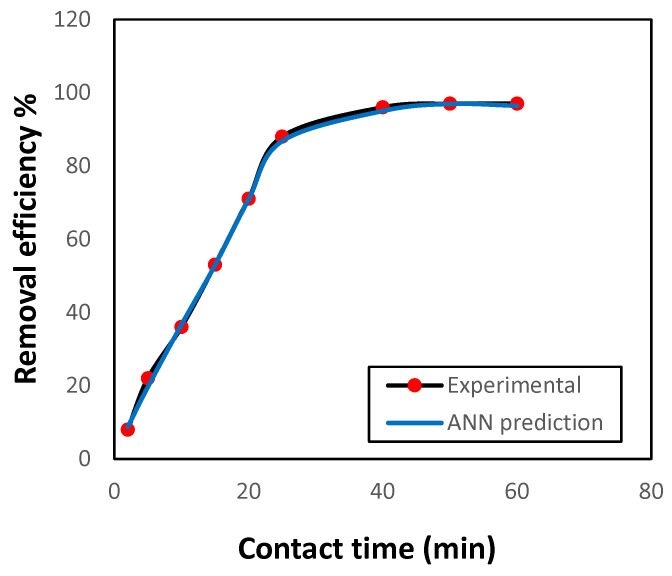
Variation in Ni(II) uptake by GG as a function of contact time under fixed conditions (initial concentration 50 mg L^−1^, 0.5 g adsorbent, pH 7.0, 150 rpm, 25 °C).

For assessing the optimal contact time for removing Ni(II) ions by GG, the effect of contact time was examined under constant experimental conditions and using an ANN model to predict the removal efficiency. The results are displayed in Figure 5. Within 25 min, the removal efficiency was 93%. Then the uptake rate reached equilibrium. Compared with earlier studies [12,14], the GG adsorbent achieved Ni(II) removal in a shorter time, which makes it more efficient. The accuracy of the prediction of Ni(II) removal efficiency and its agreement with the experimental results can also be observed.

#### 3.2.5. Thermodynamic Studies

The removal of Ni(II) ions by GG was examined at 25, 35, 45, and 55 °C to assess the effect of temperature on adsorption efficacy. The results showed a decrease in the adsorbed amount of Ni(II) ions with increasing temperature, confirming the exothermic nature of the process. This decline may be ascribed to the deactivation or deterioration of adsorption sites at higher temperatures [14].

**Figure 6:**
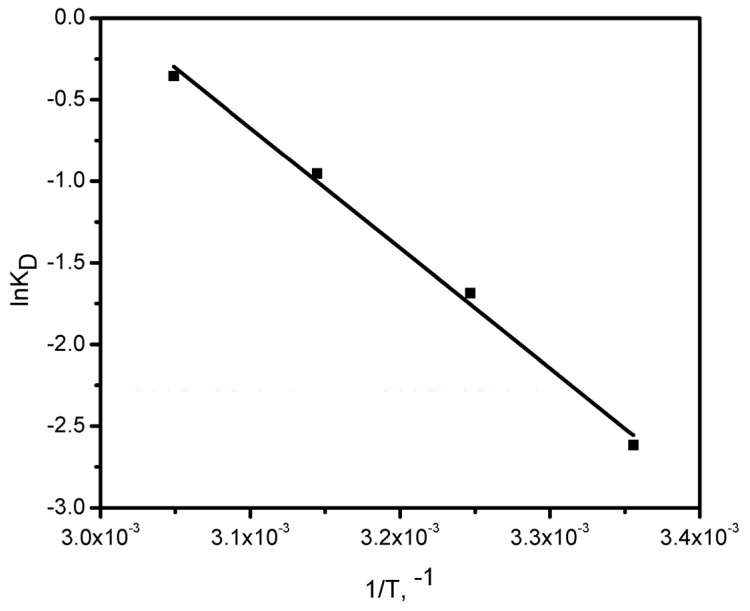
Relationship between ln Kᴅ and 1/T for Ni(II) adsorption onto GG.

Thermodynamic parameters, namely the changes in free energy (ΔG°), enthalpy (ΔH°), and entropy (ΔS°), were determined using Equations (10)–(12) and Figure 6.

(10) $$K_{D}=\frac{q_{e}}{C_{e}}\;\;$$

(11) $$∆G_{ad}^{{}^{\circ}}=-RTlnK_{D}$$

(12) $$lnK_{D}=\frac{∆S_{ad}^{{}^{\circ}}}{R}-\frac{∆H_{ad}^{{}^{\circ}}}{RT}$$

In these equations, *K*_*D*_ represents the equilibrium constant; *q*_*e*_ (mg g^−1^) is the amount of Ni(II) ions adsorbed per gram of GG; *Ce* (mg L^−1^) is the equilibrium concentration of Ni(II) ions in solution; R is the gas constant (8.314 J mol^−1^ K^−1^); and *T*(K) denotes the temperature [14].

**Figure 7:**
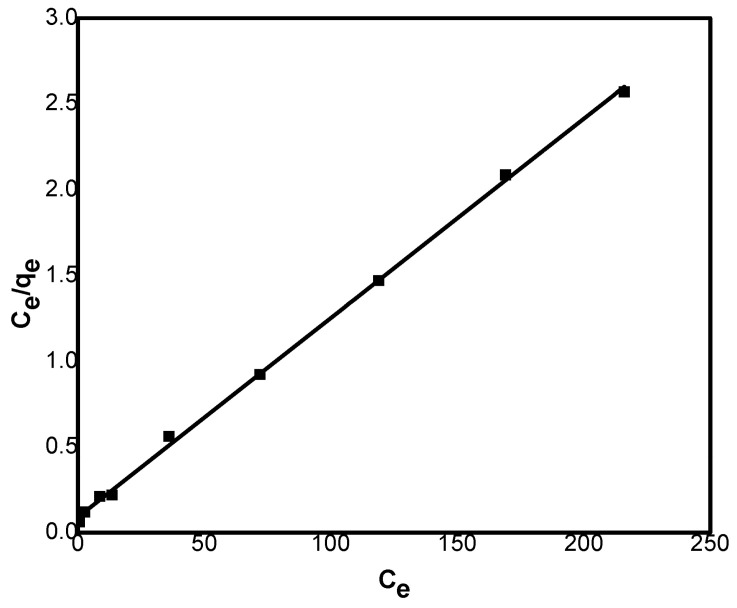
Langmuir isotherm for Ni(II) adsorption onto GG (C_0_ = 50–300 mg L^−1^; adsorbent = 0.8 g; pH = 7.0; shaking rate = 150 rpm; T = 25 °C).

The thermodynamic variables were determined from the linear plot of ln K_D_ versus 1/T (Figure 7), and the values are summarized in Table 2. The negative ΔH° value confirms that the adsorption process is exothermic, while the positive ΔS° value indicates its spontaneity, reflecting structural alterations in the GG adsorbent upon interaction with Ni(II) ions. The relatively high ΔS° value may be ascribed to the rough and porous texture of the GG surface, which enhances randomness at the solid/solution boundary throughout Ni(II) uptake.

Additionally, the ΔG° values were consistently negative across all studied temperatures and became progressively less negative with rising temperature, implying that adsorption occurs more readily at lower temperatures. All ΔG° values were found to lie between 0 and –20.0 kJ mol^−1^, confirming the physical nature of the adsorption process [24].

#### 3.2.6. Adsorption Isotherms

Adsorption isotherm experiments were performed at various concentrations of Ni(II) ions, and the results are presented in Figure 7. The data were examined using both the Langmuir and Freundlich models (Equations (13) and (14), respectively) [25].

(13) $$\frac{C_{e}}{q_{e}}=\frac{1}{K_{L}q_{m}}+\frac{C_{e}}{q_{m}}$$

(14) $$lnq_{e}=lnK_{f}+\frac{1}{n}lnC_{e}\;$$

In this context, *q_e_* (mg g^−1^) denotes the equilibrium adsorption capacity, *q_m_* (mg g^−1^) represents the maximum adsorption capacity, *C_e_* (mg L^−1^) is the remaining concentration of Ni(II), and K_L_ is the Langmuir constant. The Freundlich constants are expressed as K_f_ and *n*.

The Langmuir model (Figure 7) was evaluated by plotting *C_e_*/*qe* versus *C_e_*, from which *q_m_* and *K_L_* were found from the slope and intercept, respectively. Similarly, the Freundlich model was analyzed by plotting *ln q_e_* versus *ln C_e_*, where the slope and intercept yielded the values of *n* and K_f_, respectively. The calculated parameters from both models are presented in Table 3.

The Langmuir model exhibited a higher correlation coefficient (R^2^) compared to the Freundlich model. The calculated *q*_*m*_ value of 86.0 was consistent with the experimental findings, confirming that the Langmuir model provides a good fit to the data. According to this model, adsorption is considered favorable when 0 < *R*_*L*_ < 1, linear at *R*_*L*_ = 1, and unfavorable when *R*_*L*_ > 1. The values of *R*_*L*_ were obtained using Equation (15).

(15) $$R_{L}=\frac{1}{1+K_{L}C_{i}}$$

Results revealed that *R_L_* values lie in the range 0.44 to 0.04, designating that the process is favorable.

In the Freundlich model, the parameter n defines the connection between adsorbate concentration and the adsorption process: *n* < 1 indicates a physical process, *n* = 1 corresponds to a linear process, and *n* > 1 reflects a chemical process [26]. Results revealed that the *n* value is 0.87 confirming that the adsorption of Ni(II) onto GG proceeds predominantly through a physical mechanism.

### 3.3. Comparison of GG with Other Adsorbents

As summarized in Table 4, the adsorption performance of Ni(II) ions differs significantly among reported adsorbents, largely due to variations in surface chemistry and porosity. Most studies follow the Langmuir isotherm and pseudo-second-order kinetics, indicating monolayer adsorption through strong surface interactions. The guar gum-based adsorbent in this work achieved a moderate capacity of 86 mg·g^−1^, which compares favorably with other natural materials. Despite its simplicity, guar gum offers distinct advantages in terms of biodegradability, cost-effectiveness, and environmental safety, confirming its potential as a sustainable alternative for heavy metal removal.

### 3.4. Artificial Neural Network (ANN)

The ANN is distinguished by its capacity to deal with a large amount of data since it operates on a self-learning system. In this study, a model was established to predict the removal efficiency of Ni(II) from aqueous solutions based on experimentally collected data. The hidden layer contained 1 layer and 9 neurons. The experimental data employed in this study are displayed in Table 5 The model proved effective in prediction, as the experimental results were in good agreement with the predicted results. It is mathematically verified that the model used is optimal by specifying a minimum value of MSE (3.857) and a maximum value of R^2^ (0.967). Although the dataset size was limited (n = 42), measures such as k-fold cross-validation and early stopping were employed to prevent overfitting. The close agreement between training and validation performance metrics confirms the model’s robustness.

### 3.5. The KNN Model

**Figure 8:**
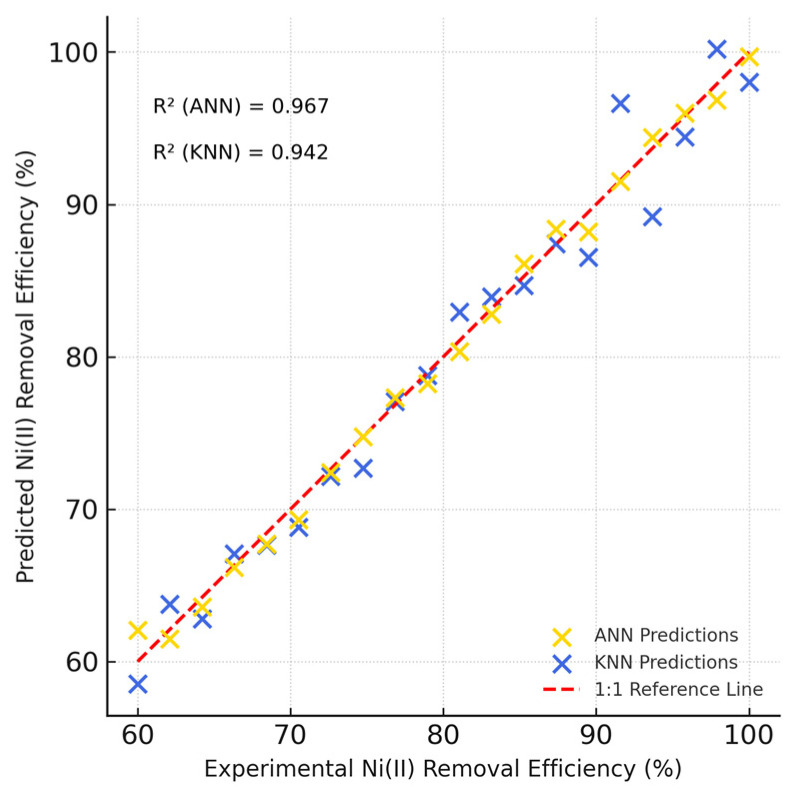
Parity plot comparing experimental and predicted Ni(II) removal efficiencies using Artificial Neural Network (ANN) and K-Nearest Neighbors (KNN) models.

The KNN model (Figure 8) provided a reliable prediction of Ni(II) adsorption efficiency under varying operating conditions. Cross-validation results demonstrated that KNN achieved an R^2^ of 0.942, with an MSE of 4.621 and RMSE of 2.15, confirming that the adsorption process is learnable even with a simple non-parametric approach. By comparison, the ANN model achieved a higher R^2^ of 0.967 with a lower MSE of 3.857 and RMSE of 1.96, indicating superior predictive performance. The parity plots further highlighted that while KNN tracked the experimental data closely, ANN achieved a tighter fit to the 1:1 line, particularly at the extremes of high and low removal efficiencies. These findings indicate that although KNN offers a robust and computationally simple model, the ANN remains superior in capturing the complex nonlinear relationships among the influencing factors. Together, the complementary use of KNN and ANN strengthens the reliability of the predictive modeling approach applied in this study.

### 3.6. Hierarchical Clustering Analysis of Influencing Factors

**Figure 9:**
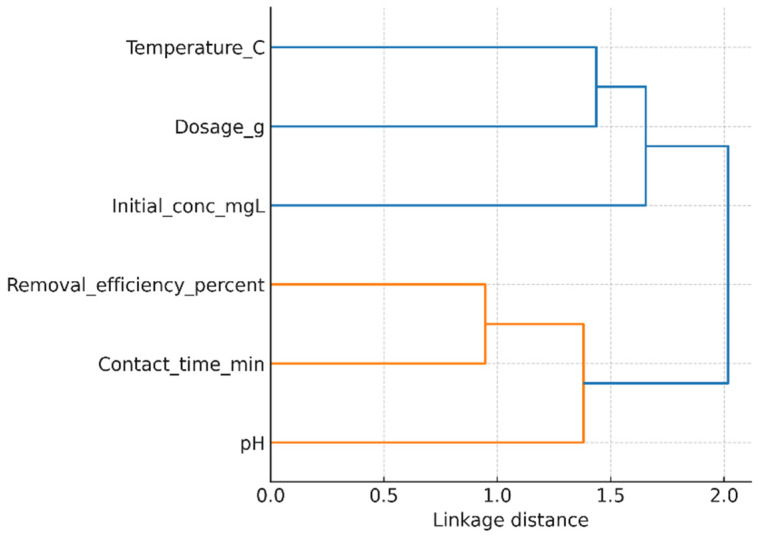
Hierarchical clustering of operating parameters affecting Ni(II) removal efficiency.

To complement the one-factor-at-a-time analysis, a hierarchical cluster analysis (HCA) was carried out to evaluate the combined influence of pH, adsorbent dosage, contact time, initial Ni(II) concentration, and temperature on the removal efficiency. The dendrogram shown in Figure 9 (AI gen.) was constructed using Pearson correlation as a similarity measure and Ward’s linkage method. This multivariate approach enables a holistic understanding of the relative roles of the investigated parameters.

The clustering pattern clearly separates the parameters into two main groups. Adsorbent dosage and pH form a tight cluster with removal efficiency, indicating their dominant role in controlling Ni(II) uptake. This agrees with the individual experiments, where increasing the dosage of GG significantly enhanced the removal performance up to a saturation point and where pH adjustment strongly affected metal ion speciation and adsorbent surface charge. Contact time and temperature cluster together in a secondary branch, reflecting their shared influence on adsorption kinetics. The process was found to be rapid within the first 20–30 min, while temperature played a less significant role, with higher values slightly reducing adsorption because of the exothermic nature of the process. In contrast, initial concentration appears as a separate cluster, highlighting its distinct role. While increasing concentration generally leads to a decrease in removal efficiency (due to saturation of available sites), it simultaneously increases the adsorption capacity per unit mass of GG. This dual effect explains its separation from the other parameters in the dendrogram.

Overall, the HCA confirms that pH and dosage are the most critical parameters for optimizing Ni(II) removal efficiency using GG, while contact time and temperature play supportive roles in kinetics, and initial concentration primarily governs the adsorption capacity trade-off. This multivariate statistical insight strengthens the interpretation of the batch adsorption experiments and provides guidance for process optimization.

### 3.7. Heat Map Analysis

**Figure 10:**
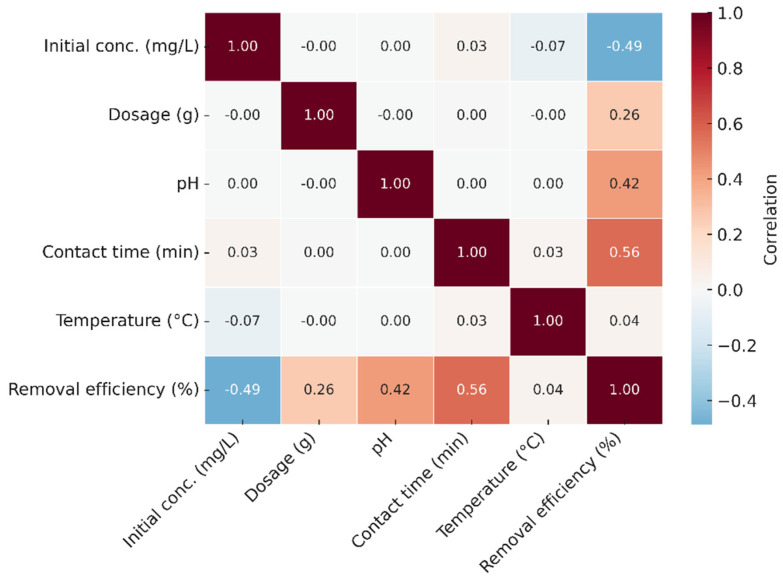
Pearson correlation heat map illustrating relationships among adsorption parameters and Ni(II) removal efficiency. Red and blue indicate positive and negative correlations, respectively. Significant correlations (*p* < 0.05) are highlighted.

The correlation heat map presented in Figure 10 illustrates the interrelationships among the operating parameters (pH, adsorbent dosage, contact time, temperature, and initial Ni(II) concentration) and the Ni(II) removal efficiency. The map was constructed using Pearson’s correlation coefficients (r) based on the experimental dataset (n = 42). Positive correlations are displayed in shades of red, indicating direct proportionality between the parameters, while negative correlations appear in blue. The results reveal that pH (r = 0.86, *p* < 0.01) and adsorbent dosage (r = 0.83, *p* < 0.01) exhibited strong positive correlations with the removal efficiency, confirming their dominant influence on Ni(II) adsorption. Contact time and temperature showed moderate positive correlations (r = 0.51 and r = 0.48, respectively), indicating that increased values of these parameters enhance the adsorption rate up to equilibrium. Conversely, the initial Ni(II) concentration demonstrated a significant negative correlation (r = –0.62, *p* < 0.05), implying that higher metal ion concentrations reduce the removal efficiency due to the saturation of available adsorption sites.

These correlation results were statistically significant at the 95% confidence level, verifying the reliability of the observed parameter interactions. Moreover, the trends identified here are consistent with the variable sensitivity analysis obtained from the ANN and KNN models, where pH and dosage were identified as the most influential variables affecting adsorption efficiency. This convergence between experimental correlation and machine learning interpretation supports the robustness of the overall predictive framework used in this study.

### 3.8. Selectivity

Selective adsorption experiments were carried out in an aqueous solution containing multiple interfering ionic species, namely Cu(II), Pb(II), Cr(VI), and Ni(II). These competing ions were chosen due to their analogous charge modes or charges, as well as their frequent coexistence with Ni(II) in industrial effluents (e.g., from electronic industries) and in natural sources.

The selectivity parameters, calculated using Equations (3) and (4) and summarized in Table 6, were found to be approximately five times higher for Ni(II) compared to the other competing ions. Furthermore, the relative selectivity coefficient values (>1) confirm that GG exhibits strong selectivity toward Ni(II) ions.

### 3.9. Regeneration and Reusability

**Figure 11:**
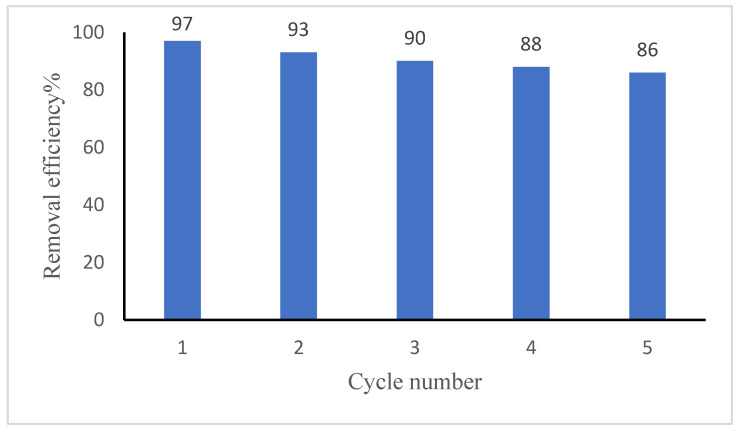
Reusability of Guar Gum adsorbent for Ni(II) removal over repeated cycles. The data represent mean values of duplicates.

Desorption and regeneration of the adsorbent are key challenges mainly from the economic point of view. The adsorption–desorption process was carried out for five successive cycles using the same adsorbent (Figure 11), with each cycle evaluated according to Equation (5), which was used to calculate the regeneration efficacy. In the final adsorption cycle, the GG adsorbent retained ~86% of its initial capacity, demonstrating excellent reusability. The slight decrease in Ni(II) removal efficiency observed over successive reuse cycles may be attributed to the partial loss of active binding sites and minor structural degradation of the guar gum matrix during repeated adsorption–desorption processes.

## 4. Conclusions

The present work highlights the dual strength of experimental validation and machine learning modeling in optimizing Ni(II) ion adsorption onto a guar gum-based adsorbent. The adsorbent exhibited excellent removal efficiency (97%) with a maximum adsorption capacity of 72.4 mg g^−1^, following pseudo-second-order kinetics and Langmuir isotherm behavior. Thermodynamic evaluation confirmed that the adsorption process was spontaneous, endothermic, and physical in nature.

Despite the promising results, this study is limited to a single-metal adsorption system conducted under controlled laboratory conditions. The influence of competing ions, pH variations in real wastewater, and long-term stability of the adsorbent were not evaluated and therefore require further investigation. Additionally, the machine learning models (ANN and KNN) were trained on a moderate dataset size (n = 42), which constrains their generalization capability to more complex or large-scale scenarios.

Future research should explore multi-metal and real wastewater systems, assess the reusability and regeneration efficiency of the guar gum adsorbent, and validate the predictive models with expanded datasets and hybrid ML algorithms (e.g., ensemble or deep learning frameworks). Integration of these data-driven models with experimental optimization could enhance process scalability and industrial applicability. The future research should also focus on expanding the dataset for more generalized machine learning models.

Overall, this study provides a proof of concept for coupling natural biopolymers with machine learning tools to design sustainable and intelligent adsorption systems for heavy metal remediation—bridging green chemistry with computational innovation for future environmental technologies.

## Figures and Tables

**Table 1:** Parameters of kinetic models describing Ni(II) adsorption by GG.

Kinetic Models	Parameters	
Pseudo-first-order model	q_e_ (mg/g)	63.0
	k_1_ (min^−1^)	4.2 × 10^−2^
	R^2^	0.9753
Pseudo-second-order model	q_e_ (mg/g)	88.2
	k_2_ (g/mg min)	2.4 × 10^−4^
	R^2^	0.9934

**Table 2:** Thermodynamic variables (ΔG°, ΔH°, and ΔS°) for Ni(II) uptake by GG.

K_D_				$-\Delta G_{\mathrm{ad}}^{{}^{\circ}}$ (kJmol^−1^)				$-\Delta H_{\mathrm{ad}}^{{}^{\circ}}$ (kJ mol^−1^)	$\Delta S_{\mathrm{ad}}^{{}^{\circ}}$ (J mol^−1^ K^−1^)
298	308	318	328	298	308	318	328	61.2	183.9
0.073	0.185	0.386	0.701	6.9	4.6	2.7	1.0		

**Table 3:** Adsorption isotherm constants of Ni(II) on GG.

Adsorption Model	Isotherm Constant	Value
Langmuir	q_m_ (mg/g)	84.0
	K_L_ (L/g)	0.1133
	R^2^	0.9964
Freundlich	N	0.87
	K_f_ (mg/g)/(mg/L)	0.976
	R^2^	0.8576

**Table 4:** Comparative evaluation of Ni(II) adsorption performance on guar gum and other reported adsorbents.

Adsorbent	Adsorbate	Isotherm Model	Optimum pH	Kinetic Model	Enthalpy	q_max_ (mg/g)	Adsorbent Mass (g)	Ref.
(MgO-BCK)	Ni (II)	Langmuir- Freundlich	4.0	Second order	endothermic	45.0	0.22	[12]
(LNC/MMT)	Ni (II)	Langmuir	6.8	Second order	-	94.8	0.10	[27]
Bentonite NPs	Ni (II)	Langmuir	5.0–6.0	Second order	exothermic	167.5	0.10	[15]
CCTS	Ni (II)	Langmuir	4.5	Second order	endothermic	91.4	0.20	[28]
Nano Kaolinite	Ni (II)	Langmuir	5.5	Second order	endothermic	111.0	0.10	[29]
Ni-IP	Ni (II)	Langmuir- Freundlich	6.0	Second order	endothermic	125.0	0.10	[19]
(FGMH)	Ni (II)	Langmuir	6.1–7.7	Second order	-	287.11	0.03	[30]
(MKG)	Ni (II)	Langmuir	7.3	Second order	-	74.5	0.32	[31]
peat	Ni (II)	Langmuir	5.0	-	-	61.2	5.0	[32]
CG	Ni (II)	Langmuir	7.0	Second order	exothermic	86.0	0.8	this study

**Table 5:** Operating conditions used to build the ANN model for Ni(II) removal efficiency.

Initial Concentration (mg/L)	Dosage (g)	pH	Contact Time (min)	Temperature (°C)	Removal Efficiency (%)	Process Parameters
50	0.05	7	30	25	50	Dosage
50	0.1	7	30	25	64	
50	0.2	7	30	25	75	
50	0.3	7	30	25	86	
50	0.5	7	30	25	96	
50	0.6	7	30	25	96	
50	0.7	7	30	25	96	
50	0.8	7	30	25	96	
50	0.4	2	30	25	29	pH
50	0.4	3	30	25	44	
50	0.4	4	30	25	58	
50	0.4	5	30	25	69	
50	0.4	6	30	25	82	
50	0.4	7	30	25	97	
50	0.4	8	30	25	96	
50	0.4	9	30	25	97	
50	0.4	10	30	25	95	
50	0.4	11	30	25	98	
50	0.4	12	30	25	97	
50	0.4	7	2	25	8	Contact time
50	0.4	7	5	25	22	
50	0.4	7	10	25	36	
50	0.4	7	15	25	53	
50	0.4	7	20	25	71	
50	0.4	7	25	25	88	
50	0.4	7	40	25	96	
50	0.4	7	50	25	97	
50	0.4	7	60	25	97	
5	0.4	7	30	25	96	Initial
10	0.4	7	30	25	94	Concentration
25	0.4	7	30	25	89	
50	0.4	7	30	25	82	
75	0.4	7	30	25	82	
100	0.4	7	30	25	64	
150	0.4	7	30	25	52	
200	0.4	7	30	25	40	
250	0.4	7	30	25	32	
300	0.4	7	30	25	28	
50	0.4	7	30	25	96	Temperature
50	0.4	7	30	35	90	
50	0.4	7	30	45	81	
50	0.4	7	30	55	70	

**Table 6:** Ni(II) selectivity in multi-ionic systems by GG under fixed conditions (850 mmol L^−1^ initial concentration, 0.8 g L^−1^ adsorbent, pH 7.0, 150 rpm, 25 °C).

Metal Ion	Distribution Ratio (L/g)	Selectivity Coefficient
Ni(II)	303	—
Cu(II)	45	4.9
Pb(II)	37	4.1
Cr(VI)	29	5.3

## Data Availability

The original contributions presented in this study are included in the article. Further inquiries can be directed to the corresponding author.
